# Cigarette Smoking, Smoking Cessation, and Bladder Cancer Risk: A Pooled Analysis of 10 Cohort Studies in Japan

**DOI:** 10.2188/jea.JE20220085

**Published:** 2023-11-05

**Authors:** Hiroyuki Masaoka, Keitaro Matsuo, Isao Oze, Takashi Kimura, Akiko Tamakoshi, Yumi Sugawara, Ichiro Tsuji, Norie Sawada, Shoichiro Tsugane, Hidemi Ito, Keiko Wada, Chisato Nagata, Tetsuhisa Kitamura, Ling Zha, Ritsu Sakata, Kotaro Ozasa, Yingsong Lin, Tetsuya Mizoue, Keitaro Tanaka, Sarah Krull Abe, Manami Inoue

**Affiliations:** 1 Division of Cancer Epidemiology and Prevention, Department of Preventive Medicine, Aichi Cancer Center Research Institute, Nagoya, Japan; 2 Department of Urology, Kyushu Central Hospital, Fukuoka, Japan; 3 Department of Epidemiology, Nagoya University Graduate School of Medicine, Nagoya, Japan; 4 Department of Public Health, Hokkaido University Graduate School of Medicine, Sapporo, Japan; 5 Division of Epidemiology, Department of Health Informatics and Public Health, Tohoku University School of Public Health, Graduate School of Medicine, Sendai, Japan; 6 Division of Cohort Research, National Cancer Center Institute for Cancer Control, Tokyo, Japan; 7 Division of Cancer Information and Control, Department of Preventive Medicine, Aichi Cancer Center Research Institute, Nagoya, Japan; 8 Department of Epidemiology and Preventive Medicine, Gifu University Graduate School of Medicine, Gifu, Japan; 9 Division of Environmental Medicine and Population Sciences, Department of Social and Environmental Medicine, Osaka University Graduate School of Medicine, Osaka, Japan; 10 Department of Epidemiology, Radiation Effects Research Foundation, Hiroshima, Japan; 11 Department of Public Health, Aichi Medical University School of Medicine, Aichi, Japan; 12 Department of Epidemiology and Prevention, Center for Clinical Sciences, National Center for Global Health and Medicine, Tokyo, Japan; 13 Department of Preventive Medicine, Faculty of Medicine, Saga University, Saga, Japan; 14 Division of Prevention, National Cancer Center Institute for Cancer Control, Tokyo, Japan

**Keywords:** bladder cancer, cohort study, Japan, pooled analysis, smoking

## Abstract

**Background:**

Although cigarette smoking is an established risk factor for bladder cancer, assessment of smoking impact on bladder cancer in Asian populations has been hindered by few cohort studies conducted in Asian populations. Therefore, we investigated the risk of bladder cancer associated with smoking status, cumulative smoking intensity, and smoking cessation in Japan.

**Methods:**

We analyzed data for 157,295 men and 183,202 women in 10 population-based cohort studies in Japan. The risk associated with smoking behaviors was estimated using Cox regression models within each study, and pooled hazard ratios (HRs) and their 95% confidence intervals (CIs) for the incidence of bladder cancer were calculated.

**Results:**

During 4,729,073 person-years of follow-up, 936 men and 325 women developed bladder cancer. In men, former smokers (HR 1.47; 95% CI, 1.18–1.82) and current smokers (HR 1.96; 95% CI, 1.62–2.38) had higher risk than never smokers. In women, current smokers had higher risk than never smokers (HR 2.35; 95% CI, 1.67–3.32). HRs in men linearly increased with increasing pack-years. Risk decreased with increasing years of smoking cessation in men, with a significant dose-response trend. Former smokers with a duration of more than 10 years after smoking cessation had no significantly increased risk compared with never smokers (HR 1.26; 95% CI, 0.97–1.63).

**Conclusion:**

Data from a pooled analysis of 10 population-based cohort studies in Japan clearly show an association between cigarette smoking and bladder cancer risk. The risk of smokers may approximate that of never smokers following cessation for many years.

## INTRODUCTION

Incidence rates of bladder cancer (BCa) differ among regions, and are the highest in Europe.^1^ In Central and Eastern Asia, where incidence is relatively low, the highest incidence of BCa is found in Japanese men.^1^ Despite a significant decrease in incidence from 2003 through 2015, the mortality of BCa in Japan has been stable.^2^ Improving mortality may require not only the development of effective treatments for advanced BCa but also stronger primary prevention to decrease BCa incidence; in particular, primary prevention requires accurate evaluation of risk factors for BCa among ethnic groups.

Cigarette smoking is an established modifiable risk factor for BCa,^3^ with a population-attributable risk of approximately 50%.^4^ Previous meta-analyses demonstrating an association of BCa risk with smoking were mainly derived from Western populations.^5^^,^^6^ These previous meta-analyses indicated that the risk of BCa associated with smoking was lower in Asian populations than in either European or North American populations. To date, however, assessment of smoking impact on BCa risk in Asian populations has been hindered by the relatively few cohort studies in Asian populations and the small numbers of participants included in meta-analyses.^7^^–^^10^ To accurately investigate this risk in Japan, we previously conducted a systematic review of three cohort and eight case-control studies in Japanese populations.^11^ Results showed a summary relative risk of 2.14 among ever smokers compared with never smokers,^11^ which also suggests a smaller impact of smoking on BCa risk in Japanese than Western populations.

Nevertheless, because these studies categorized smoking differently and did not all provide detailed information on smoking habits, we were unable to examine the association between cumulative exposure to smoking intensity and BCa risk. In contrast, analyses of data collected by large-scale prospective studies can assess the impact of smoking using the same cut-points for cumulative smoking intensity and duration. In addition, few cohort studies in Asian populations have evaluated the impact of smoking cessation on BCa risk, although doing so could encourage policy-makers and health care workers to re-emphasize the importance of counselling for patients to quit smoking.

Here, to investigate the association between BCa risk and smoking behaviors, such as smoking status, cumulative smoking intensity, and smoking cessation, we conducted a pooled analysis of 10 population-based cohort studies in Japan.

## METHODS

### Study populations

Participant data were collected from 10 major population-based cohort studies in Japan, namely the Japan Public Health Center-based Prospective Study (JPHC-I and -II),^12^ the Japan Collaborative Cohort Study (JACC),^13^ the Miyagi Cohort Study (MIYAGI-I),^14^ the Three-Prefecture Cohort Study in Miyagi (MIYAGI-II),^15^ the Three-Prefecture Cohort Study in Aichi (AICHI),^15^ the Three-Prefecture Cohort Study in Osaka (Osaka),^15^ the Takayama Study (TAKAYAMA),^16^ the Ohsaki Cohort Study (OHSAKI)^17^ and the Life-Span Study (LSS).^18^ These cohort studies were selected in accordance with the inclusion criteria, as follows: (i) started in the mid-1980s to mid-1990s in Japan, (ii) included >30,000 participants, (iii) collected self-reported data on smoking habits, and (iv) confirmed incidence of all cancers during the follow-up period. We excluded participants who had a history of other primary cancer, had missing information on smoking, or were exposed to atomic bomb radiation of ≥100 mGy (for LSS). Selected characteristics of the participants in each study are shown in Table 1. Written informed consent was obtained from all participants in each study, and the study protocols were approved by the institutional review boards of respective study centers.

**Table 1.** Characteristics of the cohort studies in the present pooled analysis

Study	Population	Age at baseline, years	Year of baseline survey	Population size	Response rate (%) of the baseline questionnaire	Method of follow-up	For the present pooled analysis						

							Age range, years	Last follow- up year	Mean follow-up period, years	Size of the cohort		Number of bladder cancer cases	

										Men	Women	Men	Women
JPHC-I	Japanese residents of 5 public health center areas in Japan	40–59	1990	61,595	82%	Cancer registry and death certificate	40–59	2012	20.1	19,778	21,606	147	42
JPHC-II	Japanese residents of 6 public health center areas in Japan	40–69	1993–1994	78,825	80%	Cancer registry and death certificate	40–69	2012	16.8	24,606	28,213	196	66
JACC	Residents from 45 areas throughout Japan	40–79	1988–1990	110,585	83%	Cancer registry (selected areas: 22) and death certificate	40–79	2009	13.4	20,426	32,889	116	52
MIYAGI-I	Residents of 14 municipalities in Miyagi Prefecture, Japan	40–64	1990	47,605	92%	Cancer registry and death certificate	40–64	2007	16.2	18,648	16,456	109	26
MIYAGI-II	Residents of 3 municipalities in Miyagi Prefecture, Japan	40+	1984	31,345	94%	Cancer registry and death certificate	40+	1992	7.6	9,398	10,730	26	5
AICHI	Residents of 2 municipalities in Aichi Prefecture, Japan	40–103	1985	33,529	90%	Cancer registry and death certificate	40–103	2000	11.5	14,996	14,226	58	22
OSAKA	Residents of 4 municipalities in Osaka Prefecture, Japan	40–97	1983–1985	35,755	85%	Cancer registry and death certificate	40–97	2000	12.3	11,304	15,936	39	17
TAKAYAMA	Residents of Takayama city, Gifu Prefecture, Japan	35+	1992	31,552	85%	Cancer registry and death certificate	35–101	2008	13.6	13,824	14,831	116	33
OHSAKI	Residents of 14 municipalities in Miyagi Prefecture, Japan	40–79	1994	52,029	95%	Cancer registry and death certificate	40–79	2005	9.3	18,439	17,528	85	40
LSS	Atomic bomb survivors in Hiroshima and Nagasaki	46–104	1991	18,877	100%	Cancer registry and death certificate	46–104	2003	10.8	5,876	10,787	44	22

Total				501,697						**157,295**	**183,202**	**936**	**325**

AICHI, Aichi Cohort Study; JACC, The Japan Collaborative Cohort Study; JPHC, Japan Public Health Center-based prospective Study; LSS, Life-Span Study; MIYAGI, The Miyagi Cohort Study; OHSAKI, Ohsaki Cohort Study; OSAKA, Osaka Cohort Study; TAKAYAMA, Takayama Study.

### Assessment of smoking

Each of the studies collected information on smoking behavior using self-administered questionnaires at baseline. The information included smoking status, age of smoking initiation, number of cigarettes smoked per day, and age at quitting smoking. Smoking status was categorized into never, former, and current smoking. Cumulative smoking intensity was estimated in pack-years (PY), defined as the number of cigarettes smoked per day divided by 20 (a pack) multiplied by the number of years of smoking, and categorized into 0, ≤20, 20–40, or >40 PY. Years since smoking cessation were categorized into ≥10, 5–9, or 0–4 among former smokers. We restricted analyses assessing the effects of cumulative smoking intensity and smoking cessation to men because of the small number of experienced smokers among women.

### Follow-up and study outcome

Participants were followed from the baseline survey until the last date of follow-up in each study. Study outcome was defined as the incidence of BCa newly diagnosed during the study period. New-onset BCa cases were identified through population-based cancer registries and/or active patient notification from major local hospitals. BCa was coded according to the International Classification of Diseases for Oncology, Third Edition (ICD-O-3) or the International Classification of Diseases and Health Related Problems, Tenth Revision (ICD-10).

### Statistical analysis

Person-years of follow-up were counted from the date of enrollment in each study to the date of BCa diagnosis, migration from the study area, death from any cause or end of follow-up, whichever came first. Cox regression models were applied to calculate hazard ratios (HRs) and their 95% confidence intervals (CIs) for the incidence of BCa. The following two models were established: model 1 adjusted for age (continuous) and area (applicable to JPHC, JACC and LSS only), and model 2 excluded cases diagnosed within the first 3 years from enrollment to minimize reversal of cause and effect. We first calculated the study-specific HRs and then applied random-effects models to obtain pooled HR estimates. When any study identified no BCa cases for a category, we pooled HRs for the corresponding category by excluding data in that study. Dose-response relationships were assessed by incorporating PYs or years since smoking cessation into the models as continuous variables, providing HRs per increase in 1 PY or 1 year of smoking cessation (unit HRs). The JPHC study and one pooled study previously reported an association between BCa risk and smoking behaviors.^8^^,^^19^ Therefore, we updated the dataset to allow longer follow-up. Heterogeneity across studies for each category was assessed using Cochran’s Q-statistic and *I*^2^-statistic. Statistical analyses were performed using STATA 15 (Stata Statistical Software Release 15; StataCorp College Station, TX, USA). *P* values of <0.05 were considered to indicate significance.

## RESULTS

A total of 157,295 men and 183,202 women participants were collected from the 10 cohort studies. The frequency of ever smokers was 79% among men and 13% among women. During 4,729,073 person-years of follow-up, 936 men and 325 women were newly diagnosed with BCa. Men was 3.47 times more likely to develop BCa than women.

In men, the HRs of former smokers and current smokers compared with never smokers were 1.47 (95% CI, 1.18–1.82) and 1.96 (95% CI, 1.62–2.38), respectively (Table 2). In women, current smokers had significantly increased risk compared with never smokers (HR 2.35; 95% CI, 1.67–3.32), whereas former smokers had an increased risk, albeit without statistical significance (HR 1.47; 95% CI, 0.75–2.88).

**Table 2.** Hazard ratios of bladder cancer risk according to smoking status

	Smoking status			Heterogeneity			
	
	Never	Former	Current	Former		Current	
	
				*P*	I^2^, %	*P*	I^2^, %
**Men**							
Number of subjects	33,196	37,783	86,316				
Person-years	473,749	500,865	1,170,469				
Number of cases	131	246	559				
HR1 (95% CI)^a^	1.00 (Ref)	**1.47 (1.18–1.82)** ^*^	**1.96 (1.62–2.38)** ^*^	0.60	0.0	0.82	0.0
HR2 (95% CI)^b^	1.00 (Ref)	**1.47 (1.17–1.86)** ^*^	**1.92 (1.56–2.36)** ^*^	0.36	8.9	0.58	0.0

**Women**							
Number of subjects	164,690	4,415	14,097				
Person-years	2,348,839	53,219	181,932				
Number of cases	277	9	39				
HR1 (95% CI)^a^	1.00 (Ref)	1.47 (0.75–2.88)	**2.35 (1.67–3.32)** ^*^	0.94	0.0	0.79	0.0
HR2 (95% CI)^b^	1.00 (Ref)	1.70 (0.79–3.68)	**2.33 (1.58–3.44)** ^*^	0.84	0.0	0.81	0.0

CI, confidence interval; HR, hazard ratio.

^a^Model 1: adjusted for age and public health center area.

^b^Model 2: model 1 excluding early cases (within the first 3 years).

^*^P < 0.05.

As shown in Table 3, HRs increased with increasing PYs among current smokers (HR 1.55; 95% CI, 1.16–2.05 for PY ≤20, HR 1.91; 95% CI, 1.54–2.37 for PY 20–40, and HR 2.36; 95% CI, 1.88–2.95 for PY >40). On combination of former smokers and current smokers, increased bladder cancer risk was similarly observed with increased PYs (HR 1.28; 95% CI, 1.00–1.63 for PY ≤20, HR 1.77; 95% CI, 1.44–2.17 for PY 20–40, and HR 2.18; 95% CI, 1.77–2.68 for PY >40), as shown in Table 4. A linear trend between BCa risk and PYs was also seen on combination of former and current smokers (HR per 1 PY, 1.01; 95% CI, 1.00–1.02). Significant heterogeneity across studies was observed in this analysis only.

**Table 3.** Hazard ratios of bladder cancer risk according to smoking status and cumulative smoking intensity among current smokers in men

	Smoking status and cumulative smoking intensity by pack-years (PY) among current smokers					Heterogeneity					
	
	Never	Former	Current			PY ≤20		PY 20–40		PY >40	
			
			PY ≤20	PY 20–40	PY >40	*P*	I^2^, %	*P*	I^2^, %	*P*	I^2^, %
**Men**											
Number of subjects	33,196	37,783	21,974	41,303	22,937						
Person-years	473,749	500,865	298,088	572,258	298,777						
Number of cases	131	246	97	250	212						
HR1 (95% CI)^a^	1.00 (Ref)	**1.47 (1.19–1.82)** ^*^	**1.55 (1.16–2.05)** ^*^	**1.91 (1.54–2.37)** ^*^	**2.36 (1.88–2.95)** ^*^	0.80	0.0	0.74	0.0	0.60	0.0
HR2 (95% CI)^b^	1.00 (Ref)	**1.48 (1.17–1.87)** ^*^	**1.66 (1.22–2.27)** ^*^	**1.84 (1.46–2.32)** ^*^	**2.32 (1.83–2.95)** ^*^	0.92	0.0	0.63	0.0	0.43	0.2

CI, confidence interval; HR, hazard ratio; PY, pack-years.

^a^Model 1: adjusted for age and public health center area.

^b^Model 2: model 1 excluding early cases (within the first 3 years).

^*^P < 0.05.

**Table 4.** Hazard ratios of bladder cancer risk according to cumulative smoking intensity on combination of former and current smokers in men

	Cumulative smoking intensity by pack-years (PY)				Unit HR (per 1 PY)	Heterogeneity for trend	
	
	0	PY ≤20	PY 20–40	PY >40		*P*	I^2^, %
**Men**							
Number of subjects	33,248	34,102	55,156	34,192			
Person-years	474,365	476,815	753,922	432,238			
Number of cases	132	151	345	307			
HR1 (95% CI)^a^	1.00 (Ref)	**1.28 (1.00–1.63)** ^*^	**1.77 (1.44–2.17)** ^*^	**2.18 (1.77–2.68)** ^*^	**1.01 (1.00–1.02)** ^*^	<0.001	87.3
HR2 (95% CI)^b^	1.00 (Ref)	**1.38 (1.05–1.80)** ^*^	**1.79 (1.44–2.23)** ^*^	**2.14 (1.71–2.68)** ^*^	**1.01 (1.00–1.02)** ^*^	<0.001	79.9

CI, confidence interval; HR, hazard ratio; PY, pack-years.

^a^Model 1: adjusted for age and public health center area.

^b^Model 2: model 1 excluding early cases (within the first 3 years).

^*^P < 0.05.

Compared with never smokers, former smokers with a duration of smoking cessation of 0–4 years and 5–10 years showed similar HRs to current smokers (HR 1.82; 95% CI, 1.35–2.45 and HR 1.81; 95% CI, 1.32–2.50, respectively), as shown in Table 5. On the contrary, former smokers with more than 10 years of smoking cessation had no significantly increased risk compared with never smokers (HR 1.26; 95% CI, 0.97–1.63). In addition, a significant linear trend in decreased BCa risk was observed as years of smoking cessation increased. These findings were not substantially changed when BCa cases diagnosed within the first 3 years after enrolment in each study were excluded.

**Table 5.** Hazard ratios of bladder cancer risk according to years since smoking cessation in men

	Smoking status considering years since smoking cessation					Unit HR (per 1 year of Smoking cessation)	Heterogeneity for trend	

	Never	Former (years since smoking cessation)			Current			
	
		≥10	5–9	0–4			*P*	I^2^, %
**Men**								
Number of subjects	33,196	18,031	8,985	10,022	86,316			
Person-years	473,749	238,392	121,475	128,697	1,170,469			
Number of cases	131	112	58	68	559			
HR1 (95% CI)^a^	1.00 (Ref)	1.26 (0.97–1.63)	**1.81 (1.32–2.50)** ^*^	**1.82 (1.35–2.45)** ^*^	**1.96 (1.62–2.38)** ^*^	**0.98 (0.97–0.99)** ^*^	0.97	0.0
HR2 (95% CI)^b^	1.00 (Ref)	1.24 (0.93–1.65)	**1.93 (1.37–2.73)** ^*^	**1.95 (1.42–2.68)** ^*^	**1.92 (1.56–2.37)** ^*^	**0.98 (0.96–0.99)** ^*^	0.83	0.0

CI, confidence interval; HR, hazard ratio.

^a^Model 1: adjusted for age and public health center area.

^b^Model 2: model 1 excluding early cases (within the first 3 years).

^*^P < 0.05.

## DISCUSSION

This pooled analysis of 10 cohort studies in Japan showed that both smoking status and cumulative smoking intensity were positively associated with BCa risk. BCa risk increased linearly as cumulative smoking intensity increased. In addition, risk was reduced as the duration of smoking cessation increased, with a significant trend. The risk in former smokers who quit smoking for ≥10 years was comparable with that of never smokers.

A carcinogenic effect of smoking on the bladder is reasonable given that tobacco contains carcinogenic compounds, including aromatic amines and N-nitroso compounds. These compounds cause DNA damage in the form of double-strand breaks, base modification and bulky adduct formation.^4^ Our present study epidemiologically confirmed that risk of BCa is increased in association with cigarette smoking. The dose-response relationship between cumulative smoking intensity and BCa risk is consistent with the findings of previous epidemiological studies among Western populations.^20^^–^^23^

On the other hand, the magnitude of the impact of cigarette smoking differed between the Japanese and Western populations. The present HR for current smokers in Japan was similar to those in Asian populations estimated by previous meta-analyses.^5^^,^^6^ The approximately two-fold increase in risk for current smokers in Japan appears lower than the approximately three-fold increase in Western populations. The lower impact of smoking in the Japanese population might be partly explained by differences in the style of inhalation and in age at the initiation of smoking.^11^ Nevertheless, the most important determinant of the difference may be variation in carcinogen-detoxification genes between ethnic groups. Analysis of 3,942 cases and 5,680 controls with a European background found six genetic variants with significant additive gene-smoking intearaction, notably in *NAT2* and *UGT1A6*.^24^ Of the six variants, significant multiplicative gene-smoking interaction was observed only in *NAT2* (ever vs never smokers, and smoking dose). The NAT2 enzyme plays an important role in detoxifying aromatic amine carcinogens, which are known bladder cancer-related carcinogens in cigarette smoke. The *NAT2* genotypes are classified into a fast acetylation type (wild-type), slow acetylation type (mutated-type), and an intermediate acetylation type having both one fast allele and one slow allele. A meta-analysis indicated that the *NAT2* slow genotype was associated with increased risk of BCa in Asian as well as Western populations.^25^ In addition, smokers with the slow *NAT2* genotype had higher BCa risk than smokers with the fast/intermediate *NAT2* genotype.^25^ Of note, the frequency of *NAT2* genotypes differed between ethnic groups: the slow genotype accounted for 59% of individuals in Europe but was much less frequent in Northeast Asian countries (China, Japan, and Korea), accounting for 18% of individuals on average.^26^ This low prevalence of the *NAT2* slow genotype in Japan may convincingly explain the lower risk of BCa among smokers compared with Western countries.

Previous epidemoiological studies consistently showed that smoking cessation results in a decrease in BCa risk, consistent with our present findings. However, whether BCa risk in former smokers with many years of cessation returns to that of never smokers in Western populations has remained controversial.^20^^–^^23^ The recent dose-response meta-analysis by van Osch et al showed that BCa risk substantially decreased as the duration of cessation lengthened, but that former smokers remained at a 50% increased risk compared with never smokers even after 20 years of cessation.^6^ Our finding that BCa risk for former smokers who quit smoking for ≥10 years was comparable to that of never smokers is inconsistent with the findings of a dose-response meta-analysis that included only one Asian cohort study.^8^ The difference in the effect of smoking cessation between Western and Japanese populations may be attributable to differences in the prevalence of the *NAT2* genotype, as noted above. Reduced exposure to smoking-related carcinogens attributable to the more prevalent fast acetylation enzyme in Japan may allow the BCa risk of former smokers with many years of smoking cessation to approximate that of never smokers. Large-scale cohort studies in other Northeast Asian populations genetically similar to the Japanese population may support our finding.

A strength of our study is its use of pooled analysis using data from large-scale prospective cohort studies. This pooled analysis included approximately 1,000 cases diagnosed with BCa, although previous cohort studies in Asian populations included only several hundred cases. The pooled analysis including a sufficient number of Japanese participants allowed us to robustly estimate BCa risk associated with smoking behaviors using unified categories for cumulative smoking intensity and smoking cessation, although meta-analyses of published articles force the integration of non-uniform populations and categories. Primary prevention by smoking cessation is critical, because no secondary prevention for BCa has yet been established. Given that fewer than half of urology outpatients perceive smoking to be a risk factor for BCa,^27^^,^^28^ general people may be even more unaware of the BCa risk associated with smoking. Our findings also emphasize the importance of urologist counselling of patients to quit smoking as soon as possible to effectively decrease BCa risk.

Our study has several potential limitations. First, we did not consider potential confounders, such as occupational exposure, a known risk factor for BCa.^4^ However, given the low frequency of occupational exposure, the effect of confounding of occupational exposure is unlikely to be remarkable. Second, the participants in the MIYAGI-II study were slightly older generation with shorter duration of follow-up than the other cohort studies. However, the results did not change substantially when the data from the MIYAGI-II study were excluded. Third, incident rates of BCa could have been underestimated because of missing early-stage BCa cases. However, the differences in the underestimation between smokers and non-smokers were unlikely, given bladder cancer is usually detected by hematuria. Thus, if present, this misclassification would have been nondifferential, leading to underestimation of the association with smoking. Fourth, we did not consider competing risks in the present study analyses. Competing risks from death attributable to cardiovascular disease and other cancer may affect the incidence of BCa, given that smoking is associated with several health problems. The beneficial effects of smoking cessation, however, did not alter substantially when we conducted competing risk analysis based on Fine and Gray’s model,^29^ using data from the major cohorts of the present study, the JPHC-I and JPHC-II studies. Thus, we believe that the risk estimates for smoking cessation in the present study are less biased by competing risks. Fifth, the number of women smokers included in the present study was small, which restricted our analysis to the association between BCa risk and smoking status only, without analysis of cumulative smoking intensity or smoking cessation. Sixth, when additionally adjusted for PY, the similar trend of a dose-response relationship between years since smoking cessation and BCa risk has been observed, but not significant, partly because of insufficient statistical power (data not shown). Future large-scale studies should aim to more closely investigate BCa risk associated with years of smoking cessation, adjusted for potential confounders.

In conclusion, we provide evidence for an association between cumulative smoking intensity and smoking cessation in a dose-response manner in men using a pooled analysis of 10 population-based cohort studies in Japan.
